# Association Analysis of Polymorphisms in *ROCK2* with Cardiovascular Disease in a Chinese Population

**DOI:** 10.1371/journal.pone.0053905

**Published:** 2013-01-11

**Authors:** Lei Liu, Yanyan Cao, Guanglin Cui, Zongzhe Li, Jing Sun, Lina Zhang, Chen Chen, Yan Wang, Peihua Wang, Hu Ding, Dao Wen Wang

**Affiliations:** 1 Departments of Internal Medicine, Tongji Hospital, Tongji Medical College, Huazhong University of Science and Technology, Wuhan, People’s Republic of China; FuWai Hospital, Chinese Academy of Medical Sciences, China

## Abstract

**Background:**

Rho-kinase (ROCK) has been shown to play an important role in cardiovascular disease such as coronary artery disease (CAD) and hypertension. Recently, common variants of *ROCK2* have been reported to influence blood pressure, but the relationship between common *ROCK2* variants and cardiovascular disease has not been extensively studied in the Chinese population.

**Methods:**

To derive a more precise estimation of their relationship, we screened for the common variants by direct sequencing of all exons of *ROCK2*, and then we performed genetic association analyses in a CAD case–control study, including a total of 1344 cases and 1267 ethnically and geographically matched controls.

**Results:**

Unconditional logistic regression showed that no significant association between common variants in the coding region of *ROCK2* and CAD was observed in our study (for rs978906, OR = 0.92, 95% CI 0.72–1.20 and *P* = 0.63; for rs2230774, OR = 0.90, 95% CI 0.70–1.16 and *P* = 0.47; for rs56304104, OR = 0.97, 95% CI 0.70–1.31 and *P* = 0.83; respectively).

**Conclusions:**

The relationship between the *ROCK2* polymorphisms and cardiovascular disease risk cannot be entirely discounted and warrants further evaluation in a large population.

## Introduction

Cardiovascular disease, in particular, coronary artery disease (CAD), is the leading cause of death and disability worldwide [1], [2]. It is caused by multiple genetic and environmental factors, and interactions among these factors [3]. Identification of susceptibility genes for cardiovascular disease is expected to provide insights into the disease pathogenesis. Recently, several candidate genes have been linked to cardiovascular disease susceptibility through genome-wide association studies (GWASs). However, the contribution of genetic risk to cardiovascular disease is still incompletely understood [4].

It is well known that Rho/Rho-kinase are thought to participate in a wide range of fundamental cellular functions, such as cell morphology, motility, contraction, adhesion, migration, proliferation, differentiation, and apoptosis [5], [6]. Rho-kinase, which belongs to serine/threonine kinase and is activated by the small G protein RhoA, was reported to play an important role in the various processes of atherosclerosis including endothelial dysfunction, inflammation and vascular smooth muscle cell proliferation [7]–[11]. Multiple animal studies have demonstrated that Rho-kinase is a critical contributor to many steps of the inflammatory atherosclerotic process and Rho-kinase inhibitors lead to up regulate of eNOS, decrease vascular inflammation and reduce atherosclerosis plaque formation [12]–[14].

Rho-kinase has two 2 isoforms: ROCK1 (chromosome loci 18q11) and ROCK2 (chromosome loci 2p24), which are assumed to be functionally redundant. ROCK1 and ROCK2 appear to be widely expressed, with ROCK2 most abundant in smooth muscle and heart, suggesting an important role of this isoform in the pathogenesis of hypertension, atherosclerosis and cardiovascular disease [15]–[17]. Previous studies have demonstrated that the Rho/Rho-kinase is increased in hypertensive animal models and hypertensive patients [18]–[21]. Recent study has identified potential hypertension susceptibility regions on chromosomes 2p24 by genome-wide linkage scan [22]. Moreover, there have been reports that single nucleotide polymorphisms (SNPs) and haplotype of *ROCK2* gene are associated with genetic susceptibility for general hypertension [23], [24]. What is more, ROCK2 is essential in inhibiting blood coagulation and maintaining blood flow in the endothelium-free labyrinth layer and that loss of ROCK2 leads to thrombus formation, placental dysfunction, intrauterine growth retardation, and fetal death [25]. In addition, Rho-kinase inhibitors can contribute to the clinical benefits in cardiovascular diseases including CAD, coronary artery spasm and hypertension [21], [26]–[28]. Since Rho/Rho kinase pathway has an important role for the pathogenesis of cardiovascular disease and atherosclerosis, the variants of *ROCK2* may influence the body’s susceptibility to cardiovascular disease.

Accordingly, we screened for the common variants in the coding sequence of *ROCK2* gene and assessed the association of common variants with CAD and blood pressure in a Chinese Han population. Additionally, a cross-sectional study on the association between the functional variants of *ROCK2* gene and the severity of CAD has been conducted.

## Methods

### Study Population and Data Collection

#### Case-control study

All participants were of Han Chinese ancestry residing Wuhan area and underwent standard medical history and physical evaluations. The selection criteria, clinical and biochemical characteristics of the study participants were described in detail in our previous report [29]. The CAD patients were consecutively recruited from the Tongji Hospital in Wuhan (Hubei, China) between May 2004 and September 2010. A case was defined as patients with angiographically confirmed narrowing of the coronary vessels by more than 50%, fatal/nonfatal myocardial infarction and/or bypass surgery. Patients with congenital heart disease, cardiomyopathy, valvular heart disease, and renal or hepatic disease were excluded from the study. The control subjects, residing in the same communities as the cases, were determined to be free of CAD and peripheral atherosclerotic arterial disease by medical history, clinical examinations, and electrocardiography. In addition, to assess the effects of *ROCK2* polymorphisms on blood pressure, we only investigated the association between SNPs and blood pressure in healthy control subjects. The control subjects who were undergoing anti-hypertensive therapy or drug treatments were excluded from the study.

#### Angiography

Subjects (n = 988) with angiographically documented CAD were included for this study. CAD cases who were previously preformed coronary artery bypassed graft surgery and undergone percutaneous intervention for a lesion for which the degree of stenosis were excluded. The coronary angiograms were reviewed by two independent angiographers who were both blinded to the results of the genotype. The severity of CAD was assessed both by the number of diseased vessels and Gensini scores. Modified Gensini scores were derived by a method previously described [30]. Each stenosis score is multiplied by a factor, which is assigned to each coronary segment depending on vessel size and importance. In each segment, the narrowing of the coronary artery lumen is rated 1 for 0–25% stenosis, 2 for 26–50%, 4 for 51–75%, 8 for 76–90%, 16 for 91–99%, and 32 for 100%. Each patient’s Gensini index is the sum of the total weights for each segment.

All the study protocols were approved by the institutional review board of Tongji hospital and informed written consent was obtained from all participants.

### DNA Isolation and Variation Screening

Fasting venous blood was collected in 5 ml EDTA tubes, and genomic deoxyribonucleic acid (DNA) was extracted from peripheral leukocytes, using a commercially available DNA isolation kit (DB-S; Fujifilm Corporation, Life Science Products Division, Tokyo, Japan), according to the protocol provided by the manufacturer (Fujifilm Corporation). Due to high (>75%) GC content of the first exon, we sequenced all other exons of *ROCK2* in 48 randomly selected individuals from our controls. Fluorescent dye-terminator cycle sequencing was performed and products were analyzed with an Applied Biosystems 3130×l capillary sequencer (Foster City, California, USA). Finally, we used the chromas program (Technelysium Pty Ltd., Helensvale, Queensland, Australia) to identify SNPs and further confirmed these SNPs by reamplifying and resequencing from the opposite strand.

### Genotyping

All SNPs were genotyped according to standard TaqMan allelic discrimination assay as previously described [31]. Universal PCR Master Mix was obtained from Applied Biosystems (Carlsbad, CA, USA). Probe and primer sequences for all SNPs were designed and synthesized by Jikang Biotech Company, Limited (Shanghai, China; Table 1). All fluorescent probes were TaqMan MGB probes consisting of a MGB modifying group, a 5′-reporter dye and a 3′-non-fluorescent quencher. All assays were conducted blindly without the knowledge of case or control status. Allelic discrimination was measured automatically using the Sequence Detection Systems 2.1 software (autocaller confidence level 95%). In addition, a total of 10% of all genotypes were repeated in independent reactions to check for consistency and to ensure genotype quality control. The results were 100% in agreement with the initial genotyping results.

**Table 1:** Sequence of probes and primers sets or relative order information.

rs ID	Primer sequences (5′→3′)	Allele	Allelic probes (5′→3′)
rs978906			
Foward	CTTTTCCATAAAGCTCTCTCGGC	A	FAM-ACACTACAATGCACACAA-MGB
Reverse	CCTCTCTGTTGAAGCTAGAAAAGATGTT	G	HEX-CACACTACAGTGCACACA–MGB
rs2230774			
Foward	TGTATACGTACTTCATTTTTCCTTGATTG	C	FAM-ATGGAATCAGTTTCTCTAC-MGB
Reverse	TGAGAATAACTTGAATAAAACAGCACATAG	A	HEX-ATGGAATCATTTTCTCTAC-MGB
rs56304104			
Foward	GAAATGATAAAAAGATCAGATTCTGCCT	A	FAM- ATTATGGCTTTTGCCAAT-MGB
Reverse	AAGTTAGTATGCTTACCTGAACCACCC	G	HEX-TATTATGGCCTTTGCCAAT–MGB

Abbreviations: FAM, 6-carboxyfluorescein; HEX, hexachloro-6-carboxyfluorescein; MGB, minor groove binder probe.

### Statistical Analysis

Continuous variables were compared between groups by univariate analysis of variance. Categorical values were compared by the χ2 test or Fisher’s test when appropriate. The distributions of genotype for variants were analyzed for deviation from Hardy-Weinberg Equilibrium (HWE) using χ2 analysis. Haploview software version 4.0 (Daly Lab at the Broad Institute, Cambridge, MA, USA) was used to assess linkage disequilibrium (LD) between SNPs. Unconditional logistic regression analysis was used to calculate ORs, 95% CIs and corresponding *P* values, after adjusting for age, sex, body mass index (BMI), hypertension, diabetes, hyperlipidemia, smoking status, glucose, family history of cardiovascular diseases and renal function as covariates. Association analyses were performed applying different models of inheritance (dominant, recessive, additive). Multiple linear regression analysis was used to test for association between SNPs and blood pressure levels in the healthy control subjects only, adjusting for age, sex, BMI and smoking status.

For CAD cross-sectional study, Gensini scores showed a markedly skewed distribution toward high values and were presented as medians and quartiles, and categorical variables were presented as frequencies with percentages. The nonparametric Kruskal-wallis test was used to compare continuous variables, and trend χ2 test and χ2 test were used for categorical variables.

Power calculation was performed by Quanto software version 1.2.3 (University of Southern California, Los Angeles, CA, USA). Assuming a minor allele frequency of 0.10 and disease prevalence of 0.5–1%, we had 80% power to detect genetic effects at an OR of 1.30, 1.32 and 2.48 under an additive, dominant, and recessive model in our samples, respectively. Statistical and association analyses were performed using SPSS 15.0 (SPSS Inc., Chicago, Illinois, USA). All tests were two-sided and *P* values less than 0.05 were considered statistically significant.

## Results

### Characteristics of Study Population

The general characteristics of the CAD case-control study population was reported in our previous report and shown in Table 2 [29]. The case-control cohort contains 1344 CAD cases and 1267 ethnically and geographically matched controls. As expected, the cases generally had higher TC, LDL-C, fasting glucose, creatinine, blood urea nitrogen and uric acid levels and lower HDL-C than that in controls. The frequencies of classical risk factors for CAD, such as hyperlipidemia, hypertension, diabetes mellitus, alcohol consumption, smoking status and family history of CAD in the patients were significantly higher than in the controls. The subjects were all of self-reported Chinese Han population.

**Table 2:** General characteristics of CAD patients and controls.

Variable	Controls	Cases
N (male %)	1267 (55.2%)	1344 (76.7%)*
Age (years)	60.5±8.5	60.4±10.2
BMI (kg/m^2^)	24.4±3.4	24.4±3.2
TC (mmol/l)	4.22±1.08	4.37±1.07*
TG (mmol/l)	1.79±2.08	1.84±1.27
HDL-C (mmol/l)	1.16±0.72	1.05±0.28*
LDL-C (mmol/l)	2.40±0.98	2.47±0.87*
FG (mmol/l)	4.9±1.7	6.4±2.9*
BUN (mmol/l)	5.8±2.4	6.2±2.1*
Cr (umol/l)	73.9±22.5	79.1±24.7*
UA (umol/l)	327.3±98.5	351.5±102.3*
Hyperlipidemia (%)	20.8	31.6*
Hypertension (%)	26.8	57.1*
Diabetes mellitus (%)	6.3	19.3*
Smokers (%)	32.8	56.3*
Drinking (%)	26.5	40.4*
Family history of CAD (%)	3.6	7.5*

Abbreviations: BMI, body mass index; TC, total cholesterol; TG, triglyceride; HDL-C, high-density lipoprotein cholesterol; LDL-C, low-density lipoprotein cholesterol; FG, fasting glucose; BUN, blood urea nitrogen; Cr, creatinine; UA, uric acid.

* *P*<0.01 cases vs. controls

### Identification and Selection of Single Nucleotide Polymorphisms

**Figure 1:**
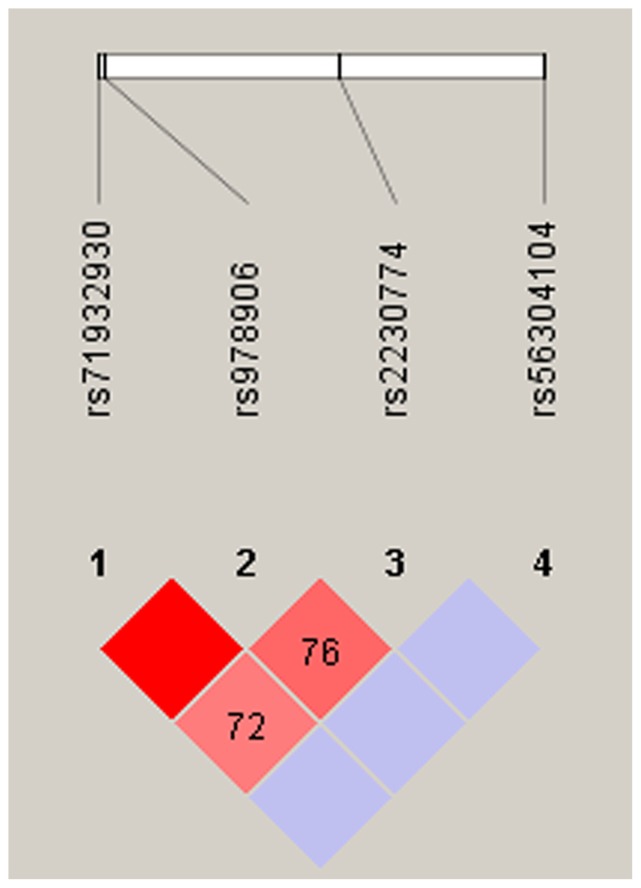
Graphical representation of SNPs in Haploview linkage disequilibrium (LD) graph of the *ROCK2* gene. Pairwise LD coefficients D′×100 were generated by Haploview 4.0 from the genotype data on 48 random controls and shown in each cell (D′ values of 1.0 are not shown). The standard colour scheme of Haploview was applied for LD colour display; logarithm of odds (LOD) score ≧2 and D′ <1, shown in pink/red; LOD score <2 and D′ <1 shown in blue/white.

Successful sequencing was achieved for 48 samples. We identified 8 polymorphisms in exons of *ROCK2*: three SNPs were in the 3′ untranslated region, three SNPs were synomnous polymorphism, and only two SNPs led to amino acid changes (Table 3). However, four of them are rare, the other four SNPs are common (minor allele frequency (MAF) >5%). On the basis of these data, we defined the linkage disequilibrium (LD) structure of the common SNPs (Figure 1) and found that the rs978906 can well capture rs71932930. Eventually, only 3 SNPs were selected for genotyping in our study.

**Table 3:** Frequency of *ROCK2* functional polymorphisms.

dbSNP	Funcation	Position^a^	Alleles	MAF
rs71932930	3′-UTR	Chr2∶11239606	−/CAAAAA	0.43
rs73915393	3′-UTR	Chr2∶11240547	G/A	0.03
rs978906	3′-UTR	Chr2∶11240727	A/G	0.41
rs2230775	Cds-synon	Chr2∶11249859	G/T	0.03
rs55839233	Cds-synon	Chr2∶11251835	T/G	0.01
rs2230774	Thr431Asn	Chr2∶11276571	C/A	0.42
rs56304104	Cds-synon	Chr2∶11307265	G/A	0.05
rs1130157	Arg83Lys	Chr2∶11344192	G/A	0.01

Abbreviations: MAF, minor allele frequency; 3′-UTR, 3′-untranslated region.

a Position is base-pair location in NCBI build 37.1 (GRCh37).

### Case-control Study Results

Table 3 shows the genotypic and allelic distributions of the three SNPs in the CAD cases and controls. The genotype frequencies of all SNPs were in accordance with HWE in the CAD group as well as in the control group (*P*>0.05). No linkage disequilibrium was found for any pair-wise combination among all three selected SNPs using Haploview 4.0 software. As shown in Table 4, the distribution of alleles and genotypes of all the SNPs were not significantly different between patient and control groups. After adjusting for age, sex, BMI, hypertension, diabetes, hyperlipidemia, smoking status, glucose, family history of cardiovascular diseases and renal function, multivariate unconditional logistic regression analysis showed that none of the three SNPs showed an association with CAD under a dominant, additive, or recessive genetic model (for rs978906, OR = 0.92, 95% CI 0.72–1.20 and *P* = 0.63; for rs2230774, OR = 0.90 95% CI 0.70–1.16 and *P* = 0.47; for rs56304104, OR = 0.97, 95% CI 0.70–1.31 and *P* = 0.83; respectively).

**Table 4:** Polymorphisms associations with coronary artery disease in our case–control study.

	Frequency		Dominant model		Additive model		Recessive model	
SNPs/genotype	Cases (n)	Controls (n)	ORs (95%CI)	*P* value	ORs (95%CI)	*P* value	ORs (95%CI)	*P* value
rs978906								
A/A	445	402						
A/G	654	620						
G/G	245	245						
MAF (%)	0.43	0.44	0.93 (0.77–1.13)	0.60	0.92 (0.72–1.20)	0.63	0.96 (0.75–1.20)	0.73
rs2230774								
C/C	444	387						
C/A	648	638						
A/A	252	242						
MAF (%)	0.43	0.44	0.87 (0.72–1.08)	0.22	0.90 (0.70–1.16)	0.47	0.95 (0.73–1.19)	0.85
rs56304104								
G/G	1224	1145						
G/A	116	120						
A/A	4	2						
MAF (%)	0.05	0.05	0.97 (0.71–1.30)	0.81	0.97 (0.70–1.31)	0.83	––	

Abbreviations: MAF, minor allele frequency; CI, confidence interval; ORs, odds ratios; ORs (95%CI) and *P* value were computed with multivariate logistic regression analysis by adjusting for age, sex, BMI, hypertension, diabetes, hyperlipidemia, smoking status, glucose, family history of cardiovascular diseases and renal function.

### Association with Blood Pressure Levels

We investigated the association between the three SNPs and blood pressure levels in 927 healthy control subjects. The control subjects who were undergoing anti-hypertensive therapy or drug treatments were excluded from the study. Results of the multiple linear regression analysis adjusted for age, gender, BMI and smoke status are shown in Table 5. After adjustment for covariates, there was no statistical correlation of the three SNPs and blood pressure levels (all *P* value >0.05).

**Table 5:** Association between *ROCK2* polymorphisms and blood pressure values in 927 health control subjects.

	SBP			DBP		
SNPs	Mean (SD)	β (SE)	*P*	Mean (SD)	β (SE)	*P*
rs978906						
A/A	123.7 (11.6)			80.7 (7.5)		
A/G	123.9 (11.7)			80.4 (7.8)		
G/G	123.7 (12.6)	0.06 (0.55)	0.92	79.8 (7.9)	−0.44(0.36)	0.22
rs2230774						
C/C	124.4 (12.5)			80.2 (8.1)		
C/A	123.6 (11.7)			80.3 (7.7)		
A/A	123.4 (11.1)	−0.51 (0.57)	0.37	80.7 (7.2)	0.22(0.37)	0.56
rs56304104						
G/G	124.0 (11.7)			80.5 (7.7)		
G/A+A/A	121.7 (13.4)	−2.30 (1.36)	0.09	79.6 (7.9)	−0.89(0.88)	0.31

Abbreviations: SBP, systolic blood pressure; DBP, Diastolic blood pressure; *P* value of the blood pressure levels was calculated by multiple linear regression model adjusted for age, sex, BMI and smoking status; A recessive model was used for rs56304104 (MAF <5%), and an additive model was used for rs978906 and rs2230774 (MAF >5%).

### Cross-sectional Study Results

We further tested the possible association between *ROCK2* polymorphisms and the severity of CAD as measured both by the number of affected vessels and Gensini scores. According to the number of significantly affected vessels, the distribution of the three SNPs genotype in different groups was presented in Table 6. By the nonparametric Kruskal-wallis test, trend χ2 test and χ2 test, there was no association of *ROCK2* polymorphisms with the severity of CAD as assessed both by the number of affected vessels and Gensini scores (all *P* value >0.05).

**Table 6:** Association of *ROCK2* with the severity of coronary artery disease.

SNPs	3VD n (%)	2VD +1VD n (%)	1VD n (%)	2VD +3VD n (%)	Gensini score
rs978906					
A/A	109 (33.6)	215 (66.4)	104 (32.1)	220 (67.9)	32.0 (15.3, 52.8)
A/G	188 (39.5)	288 (60.5)	138 (29.0)	338 (71.0)	32.0 (16.0, 56.0)
G/G	63 (33.5)	125 (66.5)	71 (37.8)	117 (62.2)	28.0 (12.3, 50.8)
Trend *P* value	0.74		0.30		
*P* value	0.16		0.08		0.31
rs2230774					
C/C	126 (39.0)	197 (61.0)	92 (28.5)	231 (71.5)	36.0 (20.0, 58.0)
C/A	172 (36.4)	301 (63.6)	154 (32.6)	319 (67.4)	30.0 (13.5, 52.0)
A/A	62 (32.3)	130 (67.7)	67 (34.9)	125 (63.1)	28.0 (13.3, 52.0)
Trend *P* value	0.13		0.11		
*P* value	0.31		0.27		0.07
rs56304104					
G/G	330 (36.5)	573 (63.5)	284 (31.5)	619 (68.5)	32.0 (16.0, 54.0)
G/A+A/A	30 (35.3)	55 (64.7)	29 (34.1)	56 (65.9)	32.0 (12.0, 54.0)
Trend *P* value	0.82		0.68		
*P* value	0.82		0.68		0.70

Abbreviations: 1VD  =  1-vessel disease; 2VD  =  2-vessel disease; 3VD  =  3-vessel disease; Variables are given as n (%) or medians (quartiles), using nonparametric Kruskal-wallis for continuous variables and trend χ^2^ test and χ^2^ test for categorical variables.

## Discussion

Cardiovascular disease is the most common cause of death worldwide and its prevalence is rapidly increasing in developing countries. The findings of many studies provide evidence for the potential role of a genetic marker in the identification of persons at risk for cardiovascular disease [32]. Despite genetic association studies have thus far identified at least 26 replicating CAD susceptibility loci of modest to small effect [33]. Their combined effect sizes explain only a minor fraction of the observed phenotypic diversity among individuals in the human population.

ROCK-dependent signaling pathway is recognized as a critical regulator of vascular functions and seems to play a central role in major cardiovascular diseases such as hypertension, atherosclerosis, and CAD. Activation of RhoA/ROCK decreases endothelial NO synthase (eNOS) expression by reducing eNOS mRNA stability and ROCKs have been shown to be upregulated at inflammatory arteriosclerotic lesions and cause coronary vasospastic responses through inhibition of myosin light chain phosphatase (MLCP) in arteriosclerotic human arteries [34], [35]. ROCKs also phosphorylate other molecules that contribute to cellular contraction, such as alpha-adducin, LIM kinases, and smooth muscle-specific basic calponin [16]. *ROCK1* and *ROCK2* appear to be widely expressed, with *ROCK2* most abundant in smooth muscle and heart, suggesting an important role of this isoform in the pathogenesis of hypertension, atherosclerosis and cardiovascular disease. Therefore, it is reasonable to postulate that *ROCK2* gene may also play an important role in the genetic susceptibility for cardiovascular disease.

For the first time, we have tested the association between CAD and multiple variations within the *ROCK2* locus using a case-control data set, and were unable to detect statistically significant association. The lack of association between the variations in the *ROCK2* gene and CAD in the current study is not totally surprising when we consider the complex pathogenesis of this disease. What is more, in the GWAS era, the *ROCK2* gene is not identified to be associated with CAD in Western or Asian population by the GWAS. It has been shown that ROCK activity contributes to the development of atherosclerosis and stenosis [36], [37]. The Gensini scoring system is a well-used method for measuring the severity of coronary atherosclerosis, which is the primary pathophysiological process underlying CAD [38]. However, we have found no significant evidence for an association between variations within the *ROCK2* locus and the severity of CAD as assessed by the number of diseased vessels and Gensini scores, which may reflect involvement of vascular mechanisms not directly related to the severity or progression of coronary atherosclerosis.

Recently, multiple lines of evidence supporting *ROCK2* in the pathogenesis of general hypertension, some studies have looked for genetic association between *ROCK2* and hypertension and have found positive results [23], [24]. However, our data do not support a major role for the three *ROCK2* SNPs in determining blood pressure levels. Consistently, another Chinese population study did not detect the association between *ROCK2* polymorphism and hypertension [39]. These inconsistent results were explained in part by population stratification, race differences, selection bias, genotyping errors, and other factors. In fact, numerous gene variants have been reported to be associated with common diseases, but few have been replicated [40]–[42]. Lack of replication has long been a big challenge in genetic association study. Considering the complex genetic network between *ROCK2* and other genes within the same pathway, the potential roles of the three SNPs might be diluted or masked by other gene–gene or gene–environment interaction. The results of long-term prospective, designed for the investigation of gene–gene and gene–environment interactions, in different ethnicity subgroups, enrolling precisely defined patients with hypertension, might produce more conclusive claims about the association between *ROCK2* and hypertension.

### Limitations

Despite being an attractive candidate gene, our genetic association analysis results do not support common variants in the coding region of *ROCK2* to have a major effect to cardiovascular disease susceptibility. However, caution should be made when interpreting the results due to some limitations of this study. Firstly, due to the exon-centric sequencing approach, we may have missed important functional variants in the non-coding regions surrounding the gene or between exons. The incomplete SNP coverage likely does not represent the entire gene and therefore may not fully describe the contribution of *ROCK2*. In the future, systematic studies of other SNPs in or adjacent to *ROCK2* in large prospective study or meta-analysis are warranted to evaluate thoroughly the role of this important gene in the genetic predisposition to cardiovascular disease. Secondly, the number of subjects examined in this study was relatively small and certainly weakened our statistical power. Thirdly, we should pay attention to the control population, as we could not exclude the possibility that some of the controls might develop cardiovascular diseases in future.

In conclusion, our results showed that allele frequency and the genotypes of the *ROCK2* polymorphisms did not change in patients with CAD. However, we cannot rule out a biological role of *ROCK2* in other populations as genetic factors influencing the pathogenesis of cardiovascular disease may differ between ethnic groups. Analysis of other variations in this gene for association with cardiovascular disease and functional studies would be helpful in elucidating the involvement of *ROCK2* gene in cardiovascular disease pathogenesis. Further validations from larger, independent populations as well as perspective studies are also required to verify these findings in different ethnic groups.
